# Transient lateral rectus palsy associated with acute coronary syndrome treated with percutaneous coronary intervention: a case report

**DOI:** 10.1186/s13256-023-04124-4

**Published:** 2023-09-13

**Authors:** Ali Asghar Farsavian, Mahya Mobinikhaledi, Parastoo Ghorbani, Erfan Ghadirzadeh

**Affiliations:** 1 Department of Cardiology, Cardiovascular Research Center, Mazandaran University of Medical Sciences, Sari, 4816117949 Iran; 2 Department of Cardiology, Faculty of Medicine, Arak University of Medical Sciences, Arak, Iran; 3 Department of Internal Medicine, Faculty of Medicine, Mazandaran University of Medical Sciences, Sari, Iran

**Keywords:** Abducens nerve disease, Acute coronary syndrome, Percutaneous coronary intervention, Complication, Case report

## Abstract

**Background:**

Patients who experience angina and acute myocardial infarction often receive diagnostic coronary angiography and percutaneous coronary intervention.

**Case presentation:**

A 54-year-old Persian man with acute coronary syndrome was admitted to the hospital and underwent elective percutaneous coronary intervention. Two hours after the procedure, the patient experienced ophthalmoplegia and diplopia. The diagnosis was abducens nerve palsy resulting in transient lateral rectus palsy. The cause is presumed to have been an ischemic event affecting the unilateral abducens nerve. This could have occurred owing to the microembolism during the percutaneous coronary intervention, which resulted in left lateral rectus palsy. Within 1 month, the diplopia was relieved completely, and the physical examination was normal.

**Conclusion:**

The occurrence of neuro-ophthalmic complications that may arise from percutaneous coronary intervention is extremely rare. To our knowledge, this is the second reported case of unilateral rectus palsy associated with percutaneous coronary intervention.

## Introduction

Myocardial infarction (MI) is a critical cardiac event with a significant burden on public health and potential consequences, such as arrhythmias and heart failure [1]. Patients who experience angina and acute MI often receive diagnostic coronary angiography (CA) and percutaneous coronary intervention (PCI) [2]. A few reports have indicated neuro-ophthalmic and retinal complications resulting from PCI or CA, which are rare complications [3–5].

Although the occurrence of neuro-ophthalmic complications that may arise from PCI is extremely rare, a variety of clinical presentations have been reported in the immediate post-PCI period [2]. Internuclear ophthalmoplegia (INO) [6], bilateral ophthalmoplegia [7], and retinopathy [8] are some reported examples of neuro-ophthalmic complications after PCI.

The paramedian pontine reticular formation (PPRF) controls the horizontal movements of the eyes, receiving information from the frontal lobe on the opposite side and transmitting it to the abducens nucleus (nVI) on the same side. The motor neurons of the abducens nucleus innervate the ipsilateral lateral rectus muscle. Furthermore, the nVI is responsible for the innervation of the contralateral medial rectus muscle by passing through the medial longitudinal fasciculus (MLF). Overall, the abducens nerve’s functions lead to the ipsilateral horizontal gaze [5]. The occurrence of isolated cranial nerve (CN) VI palsy in association with a pontine infarct is extremely rare [9]. In the majority of cases, abducens nerve palsy resulting from a lesion in the intrapontine fascicle is manifested with other neurological symptoms [10].

To the best of our knowledge, there have been only a few reports of ophthalmoplegia following diagnostic coronary angiography and percutaneous coronary intervention. In this report, we present a case of transient lateral rectus palsy resulting in diplopia in a patient who underwent coronary angiography and discuss the possible etiologies.

## Case presentation

A 54-year-old Persian man with acute coronary syndrome (ACS) was admitted to the hospital and underwent elective PCI via femoral access with stent placement in the left anterior descending artery (LAD) and obtuse marginal (OM). Contrast agent (150 cc) was injected. The blood pressure was 132/84 mmHg at time of admission. Physical examinations were normal. He took metformin sustained-release tablets t.d.s due to type 2 diabetes mellitus. Immediately after admission, sustained-release metformin tablets were discontinued. His echocardiography revealed a 45% ejection fraction with no signs of valvular dysfunction, and electrocardiogram showed normal sinus rhythm. During angioplasty, the blood pressure was 133/85 and 140/92 mmHg. Two hours after the procedure, the patient reported horizontal diplopia. His records did not show any history of problems in his eyes or nervous system. Emergency magnetic resonance imaging (MRI) and computed tomography (CT) scans were performed subsequently showed no remarkable abnormalities. The neuro-ophthalmologic evaluation revealed paresis of the lateral rectus on the left eye. There were no signs of ptosis or deficiencies in the pupillary light reflex. Other neurological examinations showed no sign suggestive of cerebrovascular accident (CVA). The patient was discharged within 48 hours after the procedure and was under observation. Within 1 month, diplopia had resolved spontaneously with normal neurological and ophthalmological examinations.

## Discussion

There are different types of brain diseases associated with cranial nerve dysfunction (including cerebral venous thrombosis, infection, subarachnoid hemorrhage, vasculitis, cerebral infarction, emergent hypertension, etc.), but the clinical presentation and radiographic findings in our case did not support their diagnosis. It has been documented that microemboli complications are the adverse effect of invasive procedures such as CA [2]. Since the incidence rate of acute stroke after PCI is 0.56%, it can be suggested that our patient experienced an ischemic event of unilateral abducens. This can happen due to microembolism during PCI resulting in left lateral rectus palsy [11]. Moreover, the proposed mechanisms of it include the formation of blood clots within the catheter and arterial wall along with their displacement. Moving the catheter may also dislodge atheromatous material, direct damage to the vessel wall by using the wire resulting in dislodging of atheromatous plaque. In PCI, the catheter itself may be responsible for dislodging plaques while entering the vessels before balloon dilation. The risk of embolic complications may be associated with prolonged and challenging procedures [12].

Contrast-induced neurotoxicity (CIN) is another plausible differential diagnosis. This rare complication has been reported in patients undergoing PCI [13]. CIN results within minutes after contrast injection and can present in different ways, including seizure, focal neurological deficit, visual loss, encephalopathy, ophthalmoplegia, and cerebellar dysfunction [14]. The proposed risk factors are renal dysfunction and injection of large volumes of contrast agents [15]. Since our patient had medical history of diabetes mellitus, he received only 150 cc of contrast agent. The impairment of cranial nerves in CIN cases is rare. In 2010, Guimaraens *et al.* reported two cases of a 71-year-old man and a 49-year-old woman who underwent vertebral and clavicle arteriography followed by aortic angiography with the involvement of cranial nerve [16]. In 1989, Lantos presented a case in which eye movement paralysis also developed in a 51-year-old man after carotid angiography [17]. Following coronary angiography, additional cases have been documented. In 2013, Vasavada *et al.* reported a case where a patient underwent angioplasty and experienced temporary unilateral partial oculomotor nerve palsy [18]. Similarly, Drummond and Wuebbolt reported a case of bilateral ophthalmoplegia in a 61-year-old woman following PCI [7]. Other instances of ophthalmoplegia after CA were reported by Yu and Dangas [19] and Caillé *et al.* [20]. Eggenberger *et al.* conducted an analysis on 110 patients with internuclear ophthalmoplegia (INO) and found that 5 of them experienced isolated INO during intravascular surgery [6]. Patients with CIN may present different radiographic findings. As initial CT scans could be normal, cerebral edema, cortical enhancement, subcortical enhancement, and hyperdensity in the subarachnoid space or parenchyma may also appear but be mistaken with intracerebral or subarachnoid hemorrhage [19, 21–23]. If a significant amount of contrast is administered, regardless of its osmolality, it can potentially disrupt the blood–brain barrier and cause neurotoxicity in the absence of preexisting brain pathology [24, 25]. Additionally, our patient experienced temporary lateral rectus palsy after undergoing PCI, which could potentially be linked to contrast-induced neurotoxicity. Extra-axial lesions including vasculopathy are also another plausible differential diagnosis for isolated abducens nerve palsy. In the majority of cases, abducens nerve palsy resulting from a lesion in the intrapontine fascicle is manifested with other neurological symptoms [10]. Thus, in such cases, it is better to perform precise examinations including MRI [26]. However, our patient had a history of diabetes mellitus and MRI revealed no signs of pontine infarction.

## Conclusion

Ophthalmological presentations associated with PCI are rare, and a variety of clinical presentations have been reported immediately post PCI. Although there are several hypotheses, the exact cause of its occurrence is unclear. Nevertheless, the most likely explanation appears to be an ischemic event of the unilateral abducens due to microembolism during PCI. The present case illustrates that PCI can result in abducens motor dysfunction. Therefore, it is important to inform the patient of the possible risks related to ophthalmoplegia and vision loss before giving their consent for the procedure.

## Data Availability

The data are available from the corresponding author on request.
